# County Hospital Reports

**Published:** 1869-08-15

**Authors:** 


### HOSPITAL REPORTS.

### *County Hospital Reports.*

A few notes made at the surgical cliniques during the present year, will convey an idea of the importance of the hospital as a school in operative surgery.

*Jan.* 5.—1. Hey's amputation of both feet. Case of *frost-bite.* Recovery with good serviceable stumps. 2. Amputation of all of the toes of both feet behind the heads of the metatarsal bones. Recovery with good stumps.

*Jan.* 12.—Case presenting the following history: Left foot frozen one year ago. Amputation through the base of the metatarsal bones. Flaps sloughed and never entirely cicatrized. On admission to the hospital a large ulcer existed upon the front of the stump, extending on one side back to the ankle. The whole stump was swollen, indurated, and in a condition which rendered impossible an attempt to save any portion of the foot. Amputation of the lower third of the leg was performed by the circular method. Recovery rapid, with a beautiful round stump. The bones of the whole tarsus were found in a state of incipient caries with exostosis and caries of the bone stump of the old operation.

*Jan.* 16.—Amputation of frost-bitten toes.

*Jan.* 25.—Lithotomy. Case of boy aged five years. Recovery.

*Jan.* 29—Amputation of frost-bitten toes.

*Feb.* 2.—Periosteal abscess at mastoid process, punctured. 2. Circumscribed peritoneal abscess. Case chronic sinus, with opening a little below umbilicus leading downward.

*Feb.* 5.—Operation for chronic hydrocele. The scrotal sack laid widely open. Evacuation of half a pint of fluid. Cavity plugged with lint saturated with a solution of carbolic acid in linseed oil. Cured.

*Feb.* 9.—1. A rare affection of the mouth. Case of papillary tumor upon inner surface of cheek, apparently like macerated epithelium in an elevated patch of slow growth, gradually spreading, giving no inconvenience. Advice, to let it alone. 2. Dressing of fractures. Plaster-of-Paris splint applied. Case of compound fracture of tibia.

*Feb.* 11.—Perineal section. Case presenting the following history: Gonorrhœa five years ago, followed by stricture, which has been practiced upon three several times by gradual dilation. When admitted to hospital there was retention of urine. A small gum catheter was introduced, after giving chloroform; a stricture was found in the bulbous portion of the urethra, which was ruptured with a Holt's dilator. In the membranous portion there was a close stricture, admitting only a No. 4 steel sound. All of the urethra posterior to this was rough and disorganized. For a time dilation was tried, but at the end of about four weeks there had been no progress. The urethra was opened freely through the perineum, and a No. 10 sound passed into the bladder. The wound healed very kindly. Advised to use the sound once in three or four weeks thereafter.

*Feb.* 19.—1. Amputation of toes of both feet. Case of frost-bite. Died of pyæmia, March 3d. 2. Amputation of leg. Case of compound Pott's fracture. Recovery.

*Feb.* 26.—Tenotomy about hip and knee .Case of inflammation of hip joint. Improved.

*March* 2.—Opening subfascial abscess of thigh.

*March* 5.—1. Amputation of middle finger. Case of traumatic gangrene. Six days ago a car wheel passed over the hand, fracturing and lacerating the index finger. In three days the little and ring fingers were gangrenous and subject to removal. A useful member preserved in remaining thumb and index. 2. Operation for removal of small subaceous tumor in supercilium.

*March* 9.—Paraceutesis of the scrotum. Case of hydrocele. Two cysts caused by adhesion between the sides of the sack.

*March* 11.—Perineal section. Case of stricture of the urethra with urinary fistulæ. Decomposed tissues and hæmorrhoids, making operation difficult. Cured.

*March* 12.—Amputation of leg. Case of caries of ankle joint following Pott's fracture.

*March* 16.—1. Injection of dilute compound tincture of iodine. Case of chronic hydrocele. Cured (March 9). 2. Amputation of great toe. Case of frost-bite. 3. Amputation of index finger. Case of crushed hand (March 5).

*March* 19.—1. Case of crushed foot, boy aged thirteen. Toes involved; tissues lacerated; bones fractured. Treatment, whisky and water—conservatives. Recovery. 2. Case of pistol-shot wound. Ball entered opposite crest of ilium, passed downward, backward, and inward. Search fruitless. 3. Case of injured knee, presenting the following history: Man aged thirty-five. Last June a car wheel struck his knee and opened the joint so that two fingers could be laid in it, patella two-thirds enucleated, bruised and ragged; a lacerated wound of the popliteal space as large as the hand. It was decided not to amputate. The patella was removed. Pus burroughed up the thigh. Swelling and pus finally disappeared. Cicatrized well; anchylosis by adhesion; position straight. Discharged from hospital at the expiration of eight months, cured. Returned the same day with rupture of the adhesions at the point of anchylosis, having met with an accident which flexed his leg upon the thigh. Leg placed at rest upon a pillow. Position, partial flexure. He will recover. 4. Removal of epulis from lower jaw.

*March* 26.—1. Dressing of fractures. 2. Operation for obliteration of varicose veins. Case of ulcer of the leg. Injection of solution of persulphate of iron. Cured.

*March* 30.—Pistol-shot wound of lumbar region. Bullet removed from a deep fluctuating abscess on side opposite wound of entrance. Large collection of pus (March 19). 2. Free openings in palm of hand to liberate burroughing pus. Case of amputated fingers, March 15 and 16).

*April* 2.—Burns. Three negroes burnt in jail. Treatment, cabon oil and lint.

*April* 6.—Railroad injury. Case of boy aged seven years. Fracture of right femur; lacerated wound of right leg; deep lacerated wound of sole of foot; compound cominuted fracture of left leg. Injury at noon. Given a teaspoonful of whisky and six drops of tincture of opium every hour. At three p. m., pale, collapsed features, sleeping. Chloroform administered; ready in four minutes; not profoundly anæsthetized. Circular amputation of the left leg two inches above the knee. Died 11 p. m.

*April* 9.—1. Compound fracture of leg, complicated with delirium tremens. 2. Epithelial execresence 'upon lower lip, fourteen years standing. Half inch lip. Base, one inch by five-eighths of an inch. Crumbling off occasionally. Excised.

*April* 16.—Death from chloroform. Case of caries of ankle joint preparing foı amputation. Middle-aged man, anœmic. Chloroform administered with a folded towel in a judicious and skillful manner. One minute after the first inspiration a convulsive movement of the extremities occurred. A few seconds later the chloroform was removed and his head declined. After four or five convulsive inspirations at long intervals, he ceased to breathe. Face livid; pupils dilated; eyes and jaws open. The heart's action was maintained for forty minutes.

*May* 4.—Operation, trepanning tibia. Case of chronic abscess. Cavity filling with new bone.

*May* 11.—Case of torticollis, from contraction of old cicatrix of a burn along the base of the lower jaw. Primary operation, division of cicatrux to secure adhesion to the bone.

*May* 24.—Operation for removal of necrosed bone. Case of gun shot; fracture of femur.

*June* 4.—Lithotomy. Case of boy aged seven years. Died of peritonitis June 10.

*June* 8.—Plastic operation. Case of torticollis. Cicatrix removed. (May 11).

*June* 13.—Operation, removal of carcinoma of the neck. Case of a man aged fifty-nine. The tumor, seventeen years in growing, had reached the size of a cocoa nut, having its origin deep on the right side near the base of the skull. An operation of the greatest difficulty performed with but little use of the knife. First, the common carotid of the affected side was ligated; then a free incision vertically through the integument. The tumor was found to have involved all the structures at the side of the neck. The mass was enucleated as far as possible by lacerating tissues. Reaching the base of the growth, it was found so firmly attached to the base of the skul and transverse processes of the superior cervical vertebræ as to require all the strength of the operator to wrest it away. The internal jugular was lacerated close to its exit from the fossa. The deluge of blood that followed ceased spontaneously. The patient reacted, but died at the expiration of the second day, with every symptom of compression of the brain. No autopsy.

*June* 25. Operation for hare-lip. Case admitted with pneumonia. Operation during convalescence. Cured. C. F.

